# Experiences with a national team-based learning program for advance care planning in pediatric palliative care

**DOI:** 10.1186/s12904-024-01515-2

**Published:** 2024-08-03

**Authors:** Marijanne Engel, Jurrianne C. Fahner, Marije P. Hennus, Consortium VIMP IMPACT, Marie-José Brounen, Carine van Capelle, Marinka de Groot, Marion Hermans, Suzanne C. Hofman, Cindy Joosen, Sarmila Lalbahadoersing-Jharap, Sofie Maebe, Erna M. C. Michiels, Suzanna Miedema, Martine F. Raphael, Jolanda Schieving, Willemien de Weerd, Marijke C. Kars

**Affiliations:** 1 Julius Center for Health Sciences and Primary Care, Center of Expertise in Palliative Care Utrecht, University Medical Center Utrecht, Universiteitsweg 100, 3584 CG Utrecht, The Netherlands; 2 Division of Pediatrics, Wilhelmina Children’s Hospital, University Medical Center Utrecht, Lundlaan 6, 3584 EA Utrecht, The Netherlands; 3 Utrecht Center for Research and Development of Health Professions Education, University Medical Center Utrecht, Utrecht, the Netherlands

**Keywords:** Advance care planning, Communication training, Learning program, Healthcare professionals, Evaluation study, Implementation, Palliative care, Pediatric care, Knowledge

## Abstract

**Background:**

Advance Care Planning (ACP) enables patients and relatives to define and share values, goals and preferences for future medical treatment and care. The IMplementing Pediatric Advance Care Planning Toolkit (IMPACT), developed in the Netherlands, is a method for conducting ACP in pediatric palliative care. Healthcare professionals who were trained to use IMPACT, indicated their need for ongoing support to practice ACP communication skills optimally over time. Therefore, we developed a team-based learning program aimed at teaching participants how to transfer knowledge on ACP, continue practicing ACP communication skills and reflect on ACP conversations within their own team context. The aim of this study was to evaluate the program’s transfer of knowledge as well as the professionals’ experience and team reflection on ACP.

**Methods:**

A one-day IMPACT train-the-trainer course was developed and a selection of healthcare professionals (facilitators) from pediatric palliative care teams (PPCTs) from all seven Dutch university hospitals and the specialized Center for Pediatric Oncology were invited to participate. Hereafter, facilitators were asked to transfer their course-acquired knowledge to their team members (learners) by organizing two coaching-on-the-job sessions. A mixed-methods design, combining questionnaires and field notes, was used to evaluate the level of knowledge transfer and team reflection achieved.

**Results:**

Eighteen healthcare professionals in the role of facilitator participated in the train-the-trainer course. In seven PPCTs one (*n* = 3) or two (*n* = 4) coaching-on-the-job session(s) took place, attended by 29 and 17 learners, respectively. In the questionnaires, 11 facilitators indicated that they had to some extent transferred acquired knowledge to their team members as intended. Sixteen out of 21 learners who participated in at least one coaching-on-the-job session, reported (somewhat) increased self-confidence for conducting ACP conversations. The reported main strength of the program was practicing with/learning from colleagues whereas dealing with workload and variation in existing ACP skills within PPCTs need more attention.

**Conclusions:**

The newly developed team-based learning program resulted in intended transfer of knowledge and methodical reflection on ACP in coaching-on-the-job sessions in most participating PPCTs. Planning coaching-on-the-job sessions regarding ACP in pediatric palliative care with multiple healthcare professionals is challenging and needs more emphasis in the training.

**Supplementary Information:**

The online version contains supplementary material available at 10.1186/s12904-024-01515-2.

## Background

In the Netherlands, yearly, around 1100 children (0–20 year) die from an underlying disease or other cause [1]. About 10.000 children with chronic conditions receive hospital and home care over a period of many years and out of them, 5000–7000 children and their families are eligible for palliative care [2]. Starting in 2012, pediatric palliative care teams (PPCTs) were developed in the seven university hospitals and in the specialized Center for Pediatric Oncology, providing integrated children’s palliative care regardless of where the child is staying [2]. In order to offer family-centered care, these PPCTs gradually give more attention to Advance Care Planning (ACP). ACP is a process that enables patients and relatives to identify and discuss values, goals and preferences for future medical treatment and care [3]. To support children, their parents and healthcare professionals in ACP, the Implementing Pediatric Advance Care Planning Toolkit (IMPACT) was developed in the Netherlands in 2019 [4, 5]. For children with life-limiting or life-threatening diseases, there is often not one “best” approach in terms of care and treatment. Parents aim for integrated care including both control of the disease and symptom management, as well as quality of life for their seriously ill child and their family as a whole [6]. Children and adolescents prefer to live their life as normal as possible. Therefore, especially in pediatric palliative care, exploring and aligning to child and family values, goals and preferences is essential. IMPACT offers a structured and concrete approach that encourages healthcare professionals to explore the perspectives of children with a life-limiting or life-threatening disease and their parents in the physical, psychological, social and spiritual domains in the now and towards the future, and to formulate values, goals and preferences for future care and treatment.

Studies show that the IMPACT approach contributes to patient-centered care and supports the process of shared decision-making [4, 7]. To facilitate implementation of IMPACT in pediatric palliative care, a two-day IMPACT training for healthcare professionals was developed. In this training, professionals learn how to conduct an actual ACP conversation based on IMPACT and practice communication skills in addition to the online available IMPACT materials [4, 5]. The training consists of lectures on the concept of ACP in pediatric palliative care and hands on communication training through role plays guided by skilled trainers and actors [4, 8].

Most professionals in pediatric palliative care acknowledge the importance of ACP, but many barriers for conducting ACP conversations with parents and children still exist [9, 10]. The transfer of knowledge and skills from a training context to clinical practice is known to be challenging and depends on several factors, such as the level of learner motivation, engagement and prior level of expertise [11]. Little is known about effective strategies for training of communication skills [12]. In preliminary evaluations, healthcare professionals who participated in the two-day IMPACT training indicated they struggle to apply the learned ACP communication skills in their daily practice while preparing and conducting ACP conversations. Also, they experience difficulties to transfer their acquired knowledge on ACP to their colleagues. Therefore, in this implementation project, a team-based learning program, consisting of a train-the-trainer course and coaching-on-the-job sessions, was developed and evaluated for its potential contribution to a sustainable implementation and dissemination of IMPACT in pediatric palliative care. Kirkpatrick’s four-level model of evaluation of training programs was used to evaluate our program [13]. This model focuses on the evaluation of different levels of transfer of knowledge: level 1 Reaction; level 2: Learning; level 3: Behavior; and level 4: Results. The aim of this project was to explore the participating healthcare professionals’ experiences with this team-based learning program and to evaluate the achieved level of transfer of knowledge and practical use of IMPACT in ACP in pediatric palliative care after introduction of this program.

## Methods

### Study design and setting

We conducted an implementation study [14, 15] using a mixed-methods design, including (open-ended) questionnaires and field notes, to evaluate how the team-based training program affected the participants’ experiences with ACP and to what level of transfer of knowledge and practical use of IMPACT in pediatric palliative care the introduction of this program led to. All eight Dutch pediatric palliative care teams (PPCTs) related to the seven university hospitals and the national Center for Pediatric Oncology, were invited to participate in this project. A PPCT is a multidisciplinary team consisting of medical, nursing, child life, psychosocial and spiritual specialists that supports children with life-limiting or life-threatening illnesses and their families [2].

### Study population

#### Participants

Healthcare professionals from each participating PPCT were selected for either the role of ‘facilitator’ or ‘learner’.

Facilitators followed the newly developed one-day train-the-trainer course and were asked to transfer their course-acquired knowledge to their team members (learners) by organizing and conducting two coaching-on-the-job sessions.

Facilitators were defined as: a) physicians or nurses or nurse practitioners working in a PPCT; b) that completed the two-day IMPACT training c) conducting ACP conversations in the context of their work and; d) willing to participate in the one-day train-the-trainer IMPACT course to be able to lead local coaching-on-the-job sessions.

Learners were defined as: a) healthcare professionals working in pediatric palliative care; b) involved in ACP conversations in the context of their work; and c) willing to be trained by a facilitator in ACP communication skills by participation in a coaching-on-the-job session.

#### Recruitment

Facilitators were invited by an open e-mail by the research team to all eight PPCTs, also inviting them for a kick-off meeting for this project. Learners were invited by facilitators of the eight participating PPCTs for participation in local coaching-on-the-job sessions between October and December 2022.

### The intervention and evaluation measures

Implementing ACP in palliative care requires a behavior change among professionals [16, 17]. Several authors argue that studies on behavior change interventions in healthcare should focus on use of diverse relevant theories to support complex real-life interventions in practice and their outcomes in healthcare [14, 15]. We developed our team-based learning program and the questionnaires prior to the official six-month study period during which the study was conducted. Both were based on insights from the IMPACT method, [4, 18] and Kirkpatrick’s four-level model [13, 19].

The team-based learning program consisted of two elements: 1) A one-day ‘train-the-trainer’ course for facilitators and 2) A coaching-on-the-job program led by facilitators for training on the use of IMPACT and reflection on actual ACP conversations in a team context in each PPCT. The existing IMPACT materials and training formed the backbone of the program [4, 5]. A detailed description of the intervention is presented in Supplemental file 1.

In order to keep the basic process in the coaching-on-the-job sessions similar for all PPCTs, facilitators used a standard presentation format with information about IMPACT and ACP for their introduction, as well as other teaching materials provided to them by the IMPACT team.

Level of transfer of training content to the participants own context was used to evaluate our program [20, 21]. Transfer refers to the targeted utilization of training-acquired knowledge and ACP communication skills by professionals in their clinical practice. We used Kirkpatrick’s four-level model of assessing training effectiveness [13, 19]. This is a widely used model, for the first time described in 1959, for evaluating training programs. The model focuses on the evaluation of: 1. Reactions: measures how participants have reacted to training activities in the team-based learning program; 2. Learning: measures what participants have learned from the train-the-trainer course or coaching-on-the-job session; 3. Behavior: measures whether what was learned is being applied on the job, i.e. the transfer of knowledge and skills to the workplace and; 4. Results: measures the occurrence of targeted outcomes. In this study, level 4, refers to the actual number of organized coaching-on-the-job sessions in PPCTs and self-reported individual results regarding practicing with and reflecting on ACP conversations in team context.

### Data collection

Data were collected by questionnaires and field notes. Facilitators received in total a maximum of four questionnaires during the study period: one questionnaire following the train-the-trainer course, one after each coaching-on-the-job session and one last questionnaire at the end of the study period. Learners also received a maximum of four questionnaires: one questionnaire at the start of the study period, one after having participated in a coaching-on-the job session and one last questionnaire at the end of the study period. Furthermore, during the study period, the researcher (ME) had close contact with the facilitators and repeatedly asked them for their intentions and actions taken to transfer acquired knowledge and skills in ACP to colleagues. If scheduling was possible, ME attended a planned coaching-on-the-job session in the role of observer. Field notes were made on all communication (via mail, phone, in person) with facilitators or learners including six coaching-on-the-job sessions ME attended [22]. A time schedule of enrolment, intervention and data collection is presented in Supplemental file 2, Table S1.

Facilitators and learners who participated in the study were assigned a study number. Data were collected in a cloud-based clinical data management system (Castor Electronic Data Capture (EDC)) through invitation emails with a personal link in order to link completed questionnaire(s) to the corresponding study number. For each uncompleted questionnaire, reminders were sent after one and two weeks post the initial invitation.

#### Questionnaires for facilitators

Questionnaires were developed based on existing literature and expert validation [23]. The first part of the first questionnaire included background characteristics. The questionnaire further focused on (i) professional’s evaluation of the train-the-trainer course (ii) acquired knowledge and skills in ACP and in team-based learning (iii) behavior in the context of conducting ACP conversations and intentions to get started with the coaching-on-the-job activities.

A second and third questionnaire were sent to facilitators who had organized and conducted a first or second coaching-on-the-job session, respectively. These questionnaires focused, on (i) a brief actual reflection on the train-the-trainer course (ii) to what extent the facilitator had taken the role of facilitator (iii) further support needed to transfer acquired knowledge and ACP skills to colleagues.

The fourth questionnaire was sent to all 18 facilitators and focused on (i) experiences with the full trajectory of the train-the-trainer course from September 2022 till January 2023 (ii) to what extent the facilitators had acted according to their plan of action prepared at the train-the-trainer course (iii) to what extent the facilitator has plans to continue practicing ACP conversations in teams after the study period. An English translation of the questionnaires for facilitators is presented in Supplemental file 3.

#### Questionnaires for learners

The first questionnaire was sent to colleague healthcare professionals, as suggested by the facilitators. The first part of the first questionnaire included questions on the learners’ background characteristics. The questionnaire further focused on (i) attitudes and beliefs towards ACP (ii) behavior in the context of conducting ACP conversations among which the part of the families to whom their PPCT provides care the learner raises the possibility of an ACP conversation.

A second and third questionnaire were sent to learners, after they had participated in a first or second coaching-on-the-job session in their team. These questionnaires focused on (i) an evaluation of the attended coaching-on-the-job session (ii) acquired knowledge and skills for ACP conversations (iii) the significance of practicing with ACP for their self-efficacy regarding ACP conversations. Learners who had not previously completed a questionnaire were first asked questions about their background characteristics and experience with ACP conversations.

The fourth questionnaire was sent to all learners who had participated in at least one coaching-on-the-job session and had filled in at least one previous questionnaire. This last questionnaire focused on (i) acquired knowledge and skills for ACP conversations ii) changed behavior due to practicing when conducting ACP conversations in their daily practice iii) to what extent the learner wants to continue practicing ACP conversations in a team setting after the study period. An English translation of the questionnaires for learners is presented in Supplemental file 4.

### Data analysis

Data were analyzed using the statistical program IBM SPSS Statistics (version 26). The results are mainly presented by descriptive statistics. Where relevant, answers to open questions in questionnaires were exported from SPSS to Word and ordered in tables. Subsequently, according to qualitative data analysis methods, answers were coded and thematically categorized, and for each open question a summary of the answers was written [24]. These codes and summaries were checked and validated by the research team. A similar analysis was performed on the field notes. Findings from the field notes were used to improve the depth of the results of the questionnaires by adding specific information to themes found where relevant, e.g., information on facilitators/barriers for organizing the coaching-on-the-job sessions as expressed by facilitators outside the questionnaires in their contacts with the researcher [25].

## Results

### Participants and training

Eighteen facilitators participated in the study. All attended the one-day ‘train the trainer’ course (see Supplemental file 1). Facilitators recruited 29 learners who participated in a first and 17 in a second local coaching-on-the-job session, of whom nine participated in two coaching-on-the-job sessions. An overview of the response rates is presented in Table 1.

**Table 1:** Response rates among facilitators and learners

	**Facilitators**			**Learners**		
	Total sent	Responses, n	Response rate, %	Total sent	Responses, n	Response rate, %
Questionnaire 1 (T1)	18	18	100.0	87	31	35.6
Questionnaire 2 (T2)	16	16	100.0	29	21	72.4
Questionnaire 3 (T3)	8	8	100.0	17	13	76.5
Questionnaire 4 (T4)	18	14	77.8	29	21	72.4

T1: For facilitators: shortly after the train-the-trainer course and for learners: at the start of the study period sent to colleague healthcare professionals, as suggested by the facilitators

T2: Shortly after the first coaching-on-the-job session

T3: Shortly after the second coaching-on-the-job session

T4: End of the study period

#### Facilitator characteristics

Of all 18 facilitators, eight were (specialized) pediatricians, nine were (specialized) pediatric nurses or nurse practitioners and one was physician assistant. Nearly all cared for ten or more children with a life-limiting illness per year. Sixteen facilitators (88.9%) had previously completed the two-day training course on IMPACT core communication skills. Two of them had completed a similar course on communication skills regarding ACP conversations (Table 2).

**Table 2:** Characteristics of facilitators and learners

		**Facilitators**	**Learners**			
			Questionnaire 1	Questionnaire 2	Questionnaire 3	Questionnaire 4
		Total (*N* = 18) N (%)	Total (*N* = 31) N (%)	Total^a,b^ (*N* = 21) N (%)	Total^b,c^ (*N* = 13) N (%)	Total^d^ (*N* = 21) N (%)
Age, years	20–30	0 (0.0)	0 (0.0)	0 (0.0)	0 (0.0)	0 (0.0)
	30–40	2 (11.1)	6 (19.4)	4 (19.0)	6 (46.2)	4 (19.0)
	40–50	9 (50.0)	10 (32.3)	7 (33.3)	3 (23.1)	7 (33.3)
	≥ 50	7 (38.9)	15 (48.4)	10 (47.6)	4 (30.8)	10 (47.6)
Sex	Female	18 (100.0)	27 (87.1)	20 (95.2)	12 (92.3)	20 (95.2)
	Male	0 (0.0)	3 (9.7)	1 (4.8)	1 (7.7)	1 (4.8)
	Not specified	0 (0.0)	1 (3.2)	0 (0.0)	0 (0.0)	0 (0.0)
Position	Physician	8 (44.4)	13 (41.9)	7 (33.3)	4 (30.8)	7 (33.3)
	Nurse	8 (44.4)	8 (25.8)	9 (42.9)	6 (46.2)	9 (42.9)
	Other	2 (11.1)	10 (32.3)	5 (23.8)	3 (23.1)	5 (23.8)
Specialty physician	Pediatrician^e^	3	9	5	4	5
	Pediatric oncologist	2				
	Pediatrician genetic and congenital disorders	1				
	Pediatric intensivist	1	2	1		1
	Pediatric neurologist	1	2	1		1
Specialty nurse	Pediatric nurse formally qualified in child care	4	7	8	4	8
	Nurse practitioner	3				
	Nurse not formally qualified in child care	1	1	1	2	1
	Nurse qualified in pediatric intensive care	1				
Other	Physician assistant	1	1		1	
	Chaplain/spiritual counselor		2	1	1	2
	Psychologist		2	1		1
	GP		1			
	Medical social worker		2	1		
	Child life specialist		2	1		1
	Pain consultant			1	1	1
Experience since licensing or graduation, years	0–5	0 (0.0)	2 (6.5)	0 (0.0)	2 (15.4)	1 (4,8)
	5–10	1 (5,6)	3 (9.7)	1 (4.8)	2 (15.4)	0 (0.0)
	≥ 10	17 (94.4)	26 (83.9)	20 (95.2)	9 (69.2)	20 (95.2)
Number of children with a life-limiting illness and their parents in care per year	< 5	0 (0.0)	3 (9.7)	0 (0.0)	0 (0.0)	0 (0.0)
	5–10	1 (5.6)	5 (16.1)	1 (4.8)	1 (7.7)	0 (0.0)
	10–20	4 (22.2)	13 (41.9)	11 (52.4)	4 (30.8)	10 (47.6)
	≥ 20	13 (72.2)	10 (32.3)	9 (42.9)	8 (61.5)	11 (52.4)
Training in field of palliative care (multiple answers possible)	Two-day IMPACT training on ACP communication skills	16 (88.9)^f^	18 (58.1)	18 (85.7)	9 (69.2)	16 (76.2)
	Other training in the field of palliative care	17 (94.4)	27 (87.1)	17 (81.0)	9 (69.2)	20 (95.0)

Total percentages for characteristics may not equal 100 due to rounding

^a^In this total, all learners that participated in a first coaching-on-the-job session in their PPCT and filled in questionnaire 2 (LEARNER version) are included. Eleven of them had not received or filled in the previous questionnaire 1 (LEARNER version)

^b^In total, 28 learners participated in one (*n* = 22) or two (*n* = 6) coaching-on-the-job sessions, and reported in total on 34 learner experiences

^c^In this total, all learners that participated in a second coaching-on-the-job session in their PPCT and filled in questionnaire 3 (LEARNER version) are included. Four of them had not filled in the previous questionnaire 1 or questionnaire 2 (LEARNER version)

^d^In this total, all learners that participated in at least one coaching-on-the-job session and filled in one of the questionnaires 1, 2 and/or 3 *and* questionnaire 4 (LEARNER version) are included

^e^Including pediatricians from specialties not given by them

^f^The two other facilitators had previously completed a similar course on ACP communication skills

#### Learner characteristics

Most learners who participated in at least one coaching-on-the-job session were 40 years or older, female, had more than 10 years working experience and cared for 10 or more children with a life-limiting per year (Table 2). Most of the learners who participated in at least one coaching-on-the-job session completed the two-day IMPACT training prior to a learner experience in the coaching-on-the-job-session(s).

### Transfer of training content

For each of the four levels of Kirkpatrick's evaluation model an overview of the most relevant answers to the closed and open questions in the questionnaires is presented for facilitators and learners respectively.

#### Level 1: Assessment of training activities: participants evaluated the train-the-trainer course and coaching-on-the-job sessions positively

Both facilitators and learners evaluated the training activities in the team-based learning program (very) positively (see Supplemental file 5, Table S1). Some points for improvement were mentioned, such as that some facilitators would have preferred a more precise indication of what was expected of them during the training program, as well as a longer study period. Some learners would have preferred more information in advance about aim and content of the coaching-on-the-job session (Table 3).

**Table 3:** Open answers: best valued elements and elements for improvement of the train-the-trainer course, coaching-on-the-job sessions and the whole team-based learning program, as experienced by facilitators and learners^a^

	^**Best valued elements (number of times mentioned)**^	^**Elements for improvement (number of times mentioned)**^
	^**Facilitators**^	
^**Train−the−trainer course**^	^−Practicing in role−plays (12x)^ ^−Involvement of a professional actor and/or the guidance by the trainer (7x)^ ^−Content, theory and/or content structure of the course (4x)^	^−More attention should be paid to the role of facilitator (6x)^ ^−The format/sentences that should be used for reflection on ACP during a coaching−on−the−job session back in their team should less explicitly be drilled (4x)^ ^−The purpose of the train−the−trainer course should be more clarified (2x)^
^**Whole team−based learning program**^	^−Practice (together) (6x)^ ^−Guidance from the research team (3x)^ ^(−For now) no change needed (6x)^	^−The time frame of the study period was too short (2x)^ ^−Other colleagues attending the one−day IMPACT training (1x)^
	^**Learners**^	
^**Coaching−on−the−job sessions**^	^−Getting the opportunity to practice (with colleagues) (15x)^ ^−Learning from observing others (13x)^ ^−Feedback received (3x)^ ^−Role−play (3x)^ ^−No change needed (8)^	^−A shorter introduction on ACP and more practicing (2x)^ ^−Provide more information on aim and content of the session in advance (2x)^ ^−More context needed for practicing in the role of another professional (1x)^
^**Whole team−based learning program**^	^−Practicing together with colleagues (10x)^ ^−Practicing in general (5x)^ ^−No change needed (12x)^	^−More detailed information about the session in advance, for example, about whether or not participants need to have followed the IMPACT course (2x)^ ^−Beyond sessions more attention to the why/how/whom of ACP in practice (1x)^

^a^Facilitators were asked for best valued elements and elements for improvement of the train-the-trainer course and the whole team-based learning program. Learners were asked for best valued elements and elements for improvement of the coaching-on the-job sessions and the whole team-based learning program. Open answers were coded to themes

#### Level 2. Learning: participants learned to use the ACP communication skills and methodically reflecting on ACP conversations from the train-the-trainer course or coaching-on-the-job sessions

All facilitators (100%) shortly following the train-the-trainer course and almost all facilitators (13 out of 14, 92.9%) at the end of the study indicated that the given information on ACP and ACP communication skills as well as the method for methodical reflection on conducting an ACP conversation in team context were clear. At the end of the study, most facilitators who filled in the last questionnaire (9 out of 14 and 8 out of 14, respectively) also indicated that, in daily practice, they were sufficiently able to transfer the ACP communication skills to colleagues in their PPCT and that they could facilitate methodical reflection on conducting ACP conversations in team context (64.3% and 57.1%, respectively) (see Supplemental file 5, Table S2).

With regard to learners, at the end of the study, 20 out of 21 learners (95.2%) (totally) agreed that the ACP communication skills were clear to them. Notably, there was a slight decrease in the relative number of learners that mentioned that they felt comfortable preparing parents for ACP from 74.2% at the start of the study to 66.7% at the end of the study as was the same for feeling comfortable conducting an ACP conversation with parents (see Supplemental file 5, Table S2).

#### Level 3 Behavior outcomes: participants applied the learned knowledge and ACP communication skills in their clinical setting

On the team level, in 7 PPCTs one or two coaching-on-the-job sessions were organized. The relative number of facilitators that indicated that they regularly reflected on their initiative with one or more colleagues on preparing for or conducting an ACP conversation increased over time from 8 out of 18 (44.4%) shortly following the train-the-trainer course, to 10 out of 14 (71.4%) at the end of the study. Furthermore, the relative number of facilitators who indicated that they raised the possibility of having an ACP conversation with half or more of the families to whom their PPCT provides care increased from 11 out of 18 (61.1%) shortly following the train-the-trainer course, to 10 out of 14 (71.4%) at the end of the study (see Supplemental file 5, Table S3). Helpful for organizing a coaching-on-the-job session were: being with two or three facilitators in a PPCT/consulting together/dividing tasks and using existing organizational structures as a preplanned multidisciplinary session. Barriers for organizing such a session were lack of time, no professional actor involved, having doubts about own skills, knowledge and especially acting abilities, lack of time due to team workload and reluctance among colleagues to role-plays.

After having participated in one or two coaching-on-the-job sessions, the relative number of learners that indicated to regularly reflect on their initiative with colleagues to prepare for an ACP conversation increased from 48.4% at the start of the study to 61.9% at the end of the study. At the end of the study, for each ACP communication skill, 7 to 12 out of 21 learners (33.3% to 57.1%) indicated that they felt more confident in this skill. However, at the end of the study, for each of these skills, compared to their feeling confident in a skill, less learners, i.e., 5 to 7 learners (23.8% to 33.3%) indicated that they actually had started to use this ACP communication skill more in ACP conversations (see Supplemental file 5, Table S3).

#### Level 4 Results in PPCT: facilitators transferred training content during coaching-on-the-job sessions resulting in half of the participating learners to report (some) positive change in their attitude and self-confidence towards ACP conversations

In this level 4, the targeted outcomes of the team-based learning program were measured. On the team level, the facilitators of 7 out of 8 PPCTs organized a *first* coaching-on-the job session in their team which was attended by a total of 29 learners (range 1 to 7). In 4 PPCTs a *second* coaching-on-the job session was organized, which was attended by 17 learners (range 2 to 6). All coaching-on-the-job sessions had a mean of 4.2 learners per session and lasted an average of 85 min per session (range 30 to 120 min). In one PPCT no coaching-on-the-job session was organized. Both at the start and end of the study, facilitators and learners were not able to give an estimation of the number of families involved in an ACP conversation by the PPCT during the past six months.

On an individual level, half of the facilitators indicated at the end of the study that they had met their preset goal: i.e., they had organized and conducted two coaching-on-the-job sessions in their PPCT. In addition, at the end of the study, 11 out of 14 (78.6%) of the facilitators expected to continue to apply the skills learned for methodical reflection in their PPCT beyond the end of the research period, and 11 out 14 (78.6%) had already scheduled another coaching-on-the-job session in their team or intended to do so (see Supplemental file 5, Table S4). Four facilitators mentioned as reasons for not having met their goals: I need more practicing, the planning of a session is difficult, in my PPCT due to a high workload there is little motivation for sessions and I don’t know.

Almost half of the learners indicated that the coaching-on-the-job session(s) they had attended changed something in their attitude and self-confidence toward conducting ACP conversations by themselves or colleagues (see Supplemental file 5, Table S4). A positive change that was mentioned most frequently in answer to an open question was: I feel more confident in conducting an ACP conversation. At the end of the study, 18 out of 21 (85.7%) learners indicated that they expected to continue to apply the ACP communication skills after the end of the research period and 15 out of 21 (71.4%) strongly intended to participate in a subsequent session for practicing ACP conversations.

Best valued elements and elements for improvement of the whole team-based learning program that facilitators and learners mentioned are presented in Table 3.

#### Field notes show enthusiasm for the program and facilitators’ need for (more) guidance

Field notes show the overall enthusiasm of most participants in the program, however most facilitators needed one or more emails and telephone calls from the research team to encourage them to organize their first coaching-on-the-job session. Field notes reveal that facilitators sometimes struggled to identify colleagues eligible for a coaching-on-the-job session, because it was not very clear to them who were involved in ACP. Furthermore, for some PPCTs it worked well to use (part of) a regular team meeting for the coaching-on-the job session. For other PPCTs this meant that if the regular patient briefing was compromised, facilitators found it difficult to find another moment for the coaching-on-the-job session due to team workload and different schedules. Apart from organizational issues some facilitators felt very unsure about their role as facilitator and assumed great resistance to role-playing in their PPCT.

With regard to the coaching-on-the-job sessions attended by the researcher (ME), field notes show that most participants actually appreciated the role-plays and indicated that in the role-play they acted as they would normally do in real conversations with parents and/or their child. Some of them also indicated that they learned a lot from observing the way colleagues conducted ACP conversations, from their use of specific sentences or words or silences. Participants also appreciated playing the role of a parent. This increased their empathy for parents and taught them a lot about clinician-parent communication, for example, about how it may appear to a parent when a professional gives a lot of information at once.

## Discussion

### Summary of findings

This study explored the experiences of healthcare professionals in pediatric palliative care with a newly team-based learning program on ACP. In addition, it evaluated to what degree this team-based learning program facilitated transfer of knowledge regarding ACP communication skills in conducting ACP conversations from a train-the-trainer course to the participants’ real-work context. Most participants rated the learning program very positively although embedding it in daily practice appeared to be challenging. ‘Facilitators’ of seven out of eight PPCTs organized and guided one or two coaching-on-the job sessions in their team and met our pre-set goal of transferring course acquired knowledge and skills on ACP communication skills to their PPCT. Of the ‘learners’ who participated in these coaching-on-the-job sessions, almost all respondents expected to continue to apply the ACP communication skills learned, during their ACP conversations with parents and/or children, beyond the end of the study period.

#### Continuous practicing of ACP communication skills and methodical reflection on ACP conversations

Even if healthcare professionals are familiar with ACP, starting and conducting an ACP conversation seems still to be difficult, often resulting in introducing ACP in a very late phase of the illness trajectory [9, 10]. Most participants in our study appreciate the (re-)introduction of theory on ACP and IMPACT, and the repeated practicing of skills in short role-plays in the newly developed training program. Hereby our results support the results of other studies that argue for continuous learning and evaluation of processes in healthcare to continuously improve care processes in general [14] and ACP in pediatric palliative care in particular [9, 10].

#### Transfer of knowledge on ACP and ACP communication skills

Knowledge transfer is known to be a dynamic process that unfolds over time, resulting from the interaction between persons, situations and criteria over time [26]. Literature shows that three major factors affect the extent of knowledge transfer to the job: 1. trainee characteristics; 2. characteristics of the training activities; and 3. work environmental factors [21, 26].

##### Trainee characteristics

Both facilitators and learners in general were very motivated to participate in learning activities aimed to optimize ACP in pediatric palliative care as is also known from the literature [9, 18]. Our results show that, although a relatively small number of participants, most of them experience positive changes in attitude and skills and (strongly) intend to continue practicing ACP communication skills in combination with methodical reflection on ACP conversations. However, knowledge transfer resulting in professionals applying learned knowledge, skills and attitude over time is known to be difficult and needs ongoing attention [26].

The fact that only one third of learners at the end of the study had started to use more (some) ACP communication skills may be explained by several reasons. Except that the coaching-on-the-job session may have not fit to their professional or personal development needs, [21] a trivial reason may be that some learners did not conduct an ACP conversation during the rather short study period of a maximum of three months between the coaching-on-the-job sessions and the final questionnaire. As is also known from other studies on ACP, healthcare professionals may find it difficult to label conversations with parents and/or children as ACP conversation [18]. Another reason for not changing their behavior may be that participants, in their own opinion, already are doing ACP conversations following a more or less well-defined method and feel that they do not have to change their behavior.

A striking finding is a slight decrease in the number of learners that at the end of the study felt comfortable in conducting an ACP conversation with parents. This finding may be explained by the fact that the participation in a coaching-on-the-job session leads to better understanding of the method and ACP communication skills required for ACP conversations or increased (self-) awareness over time and thereby to greater uncertainty about whether one is doing ACP as intended [8, 27]. This is known also as the Dunning-Kruger effect: the tendency of people with low ability—to apply skills—in a specific area to give overly positive assessments of this ability [28] as could have been the case for learners before their participation in the coaching-on-the-job sessions [28].

##### Characteristics of the training activities

Our main influence in the present study was on the second affecting factor, the development of the training activities. Besides training knowledge and ACP communication skills on the individual level, our team- based learning program focuses explicitly on team-level factors which are known to promote interdisciplinary collaboration in palliative care, [29, 30] such as discussing and reflecting on ACP conversations in team-context [22]. Facilitators and learners give overall very positive feedback on the team-level aspects of the training activities. One way to improve the quality of this team-based learning program on ACP communication skills could be to train facilitators also explicitly on the role of champions or frontrunners, who may play an important role in promoting ACP in their PPCT and beyond [31–33]. Taking a leadership role in their team may involve a great challenge for healthcare professionals [34]. Another issue for improvement in our team-based program is that the target group for the training program should be better defined. Although there has been shown high effectiveness of interprofessional training in pediatric palliative care, [30] ACP may be a too specific medical/nursing intervention to train disciplines that have a key role and disciplines that have a derived role in ACP together.

An important next step could be to assess after for example one year what team members actually conduct ACP conversations before and after implementing this program, and then to evaluate the experiences of parents and children with these conversations. Next, the findings of this follow-up studies can be integrated in the team-based learning program.

##### Work environmental factors

Most mentioned barriers to the program are found in the work environmental factors, such as having difficulties in planning coaching-on-the-job sessions due to a high team workload and different schedules. From other studies it is known that frontrunners or champions in non-specialized palliative care also have difficulties to disseminate knowledge to colleagues, or may fail to organize meetings due to e.g. a high workload or lack of dedicated time [35]. Therefore, in the future more attention should be paid to the guidance of facilitators in ways appropriate to them/their PPCT to organize coaching-on-the-job sessions and, if needed, adaptation of local coaching-on-the-job activities to the specific needs and characteristics, such as prior ACP training of professionals working in a certain institute. In addition, at the organizational and management level more importance should be given to the ongoing training of healthcare professionals on communication skills, similar to training on both medical and nursing technical skills [36].

#### Strengths and limitations of the study

A strength of the study was triangulation of data by the researcher (ME) attending some sessions leading to extra information in addition to the results of the questionnaires. Furthermore, the regular contact between the research team and the facilitators, and the observations during some sessions, were helpful in getting an overall picture of the process that was going on in the PPCTs. However, this level of intervening in the natural process could also be considered a limitation. Other limitations include the under-representation of male pediatric care professionals under the age of 40 that nonetheless represents actual pediatric care practice. Also the rather short study period of six months might have led to a large time pressure on the facilitators to organize two coaching-on-the-job sessions in a period of two or three months. In this study we found that a pro-active planning of activities guided by the research team proved to be helpful. Another limitation is that in some PPCTs the first questionnaire was distributed broadly to many types of professionals and in other PPCTs more narrowly to nurses and physicians. The same was true for the coaching-on-the-job sessions: in some PPCTs in addition to the original target group of nurses and physicians also other professionals participated in the coaching-on-the-job sessions. This sometimes led to different needs regarding theory on ACP provided by facilitators and practicing ACP during the session and to some professionals feeling not addressed by certain questions in the questionnaires. Finally, the small number of learners means that conclusions must be drawn with caution.

#### Conclusion

The newly developed team-based learning program to facilitate continuous training and reflection on the use of IMPACT seems a promising intervention for the 'transfer of knowledge' on ACP, ACP communication skills and reflection on ACP conversations in a team context. The team-based learning program may contribute to a sustainable implementation and dissemination of IMPACT in pediatric palliative care. However, for many healthcare professionals in PPCTs who regularly conduct ACP conversations, practicing ACP communication skills and reflecting on ACP does not come naturally. For methodically practicing with and reflecting on ACP in team context, PPCTs need more dedicated time for coaching-on-the-job activities related to ACP and facilitators need more guidance during these coaching-on-the-job sessions so they know how to deal with individual variation between their team members in conducting ACP.

### Supplementary Information

Supplementary Material 1.

Supplementary Material 2.

Supplementary Material 3.

Supplementary Material 4.

Supplementary Material 5.

## Data Availability

The data of this study are kept by M.C.K. in the University Medical Center Utrecht, Utrecht, the Netherlands, and are available upon reasonable request.

## References

[1] CBS StatLine. StatLine the Deceased; Major causes of death (short list), age, gender [in Dutch] 2024 [Available from: https://opendata.cbs.nl/statline/#/CBS/nl/dataset/7052_95/table?ts=1566378924628. Accessed on 20 Feb 2024.

[2] Vallianatos S, Huizinga CSM, Schuiling-Otten MA, Schouten-van Meeteren AYN, Kremer LCM, Verhagen AAE. Development of the Dutch structure for integrated children's palliative care. Children (Basel). 2021;8(9). 10.3390/children8090741 PMC8470608 34572173

[3] Rietjens JAC, Sudore RL, Connolly M, van Delden JJ, Drickamer MA, Droger M, et al. Definition and recommendations for advance care planning: An international consensus supported by the European Association for Palliative Care. Lancet Oncol. 2017;18(9):e543–51. 28884703 10.1016/S1470-2045(17)30582-X

[4] Fahner J, Rietjens J, van der Heide A, Milota M, van Delden J, Kars M. Evaluation showed that stakeholders valued the support provided by the Implementing Pediatric Advance Care Planning Toolkit. Acta Paediatr. 2021;110(1):237–46. 32434275 10.1111/apa.15370 PMC7818164

[5] Fahner JF, Kars MC. Talk about care & treatment together (about IMPACT with animation Advance Care Planning) Utrecht: University Medical Center Utrecht; 2023 [Available from: https://kinderpalliatief.nl/impact/en/.

[6] Verberne LM, Kars MC, Schouten-van Meeteren AY, Bosman DK, Colenbrander DA, Grootenhuis MA, van Delden JJ. Aims and tasks in parental caregiving for children receiving palliative care at home: A qualitative study. Eur J Pediatr. 2017;176(3):343–54. 28078429 10.1007/s00431-016-2842-3 PMC5321698

[7] Van Driessche A, Gilissen J, De Vleminck A, Kars M, Fahner J, van der Werff Ten Bosch J, et al. The BOOST paediatric advance care planning intervention for adolescents with cancer and their parents: Development, acceptability and feasibility. BMC Pediatr. 2022;22(1):210. 10.1186/s12887-022-03247-9 PMC9010242 35428281

[8] Back AL, Fromme EK, Meier DE. Training clinicians with communication skills needed to match medical treatments to patient values. J Am Geriatr Soc. 2019;67(S2):S435–41. 31074864 10.1111/jgs.15709

[9] Lotz JD, Jox RJ, Borasio GD, Führer M. Pediatric advance care planning from the perspective of health care professionals: A qualitative interview study. Palliat Med. 2015;29(3):212–22. 25389347 10.1177/0269216314552091 PMC4359209

[10] Sanderson A, Hall AM, Wolfe J. Advance care discussions: Pediatric clinician preparedness and practices. J Pain Symptom Manage. 2016;51(3):520–8. 26550935 10.1016/j.jpainsymman.2015.10.014

[11] Hajian S. Transfer of learning and teaching: A review of transfer theories and effective instructional practices. IAFOR Journal of Education. 2019;7(1):93–111. 10.22492/ije.7.1.06

[12] Bos-van den Hoek DW, Visser LNC, Brown RF, Smets EMA, Henselmans I. Communication skills training for healthcare professionals in oncology over the past decade: A systematic review of reviews. Curr Opin Support Palliat Care. 2019;13(1):33–45. 10.1097/SPC.0000000000000409 30562180

[13] Kirkpatrick D. Great ideas revisited. Training Development. 1996;50(1):54.

[14] Hulscher M, Wensing M. Process Evaluation of Implementation Strategies. In: Wensing M, Grol R, Grimshaw J, editors. Improving Patient Care: The Implementation of Change in Health Care. 3rd ed. Hoboken, USA: Wiley-Blackwell; 2020. p. 369–88.

[15] Peters DH, Adam T, Alonge O, Agyepong IA, Tran N. Implementation research: What it is and how to do it. BMJ. 2013;347:f6753. 24259324 10.1136/bmj.f6753

[16] Fried TR, Bullock K, Iannone L, O’Leary JR. Understanding advance care planning as a process of health behavior change. J Am Geriatr Soc. 2009;57(9):1547–55. 19682120 10.1111/j.1532-5415.2009.02396.x PMC2783892

[17] Oh A, Allison TA, Mahoney K, Thompson N, Ritchie CS, Sudore RL, Harrison KL. Front-line hospice staff perceptions of barriers and opportunities to discussing advance care planning with hospice patients and their families. J Am Med Dir Assoc. 2022;23(7):1205–14.e2. 34391713 10.1016/j.jamda.2021.07.014 PMC8840996

[18] Fahner JC, Rietjens JAC, van der Heide A, van Delden JJM, Kars MC. Survey of paediatricians caring for children with life-limiting conditions found that they were involved in advance care planning. Acta Paediatr. 2020;109(5):1011–8. 31625623 10.1111/apa.15061 PMC7216915

[19] Kirkpatrick JD, Kirkpatrick WK. Kirkpatrick’s Four Levels of Training Evaluation: Association for Talent Development. 2016.

[20] Chia CF, Nadarajah VD, Lim V, Kutzsche S. Transfer of knowledge, skills and confidence from a faculty development programme for health professions educators into practice. Med Teach. 2021;43(sup1):S46–52. 32552199 10.1080/0142159X.2020.1776239

[21] Yelon SL, Ford JK, Anderson WA. Twelve tips for increasing transfer of training from faculty development programs. Med Teach. 2014;36(11):945–50. 24984563 10.3109/0142159X.2014.929098

[22] Hill DL, Walter JK, Casas JA, DiDomenico C, Szymczak JE, Feudtner C. The codesign of an interdisciplinary team-based intervention regarding initiating palliative care in pediatric oncology. Support Care Cancer. 2018;26(9):3249–56. 29627863 10.1007/s00520-018-4190-5 PMC6379076

[23] Artino AR Jr, La Rochelle JS, Dezee KJ, Gehlbach H. Developing questionnaires for educational research: AMEE Guide No. 87. Med Teach. 2014;36(6):463–74. 24661014 10.3109/0142159X.2014.889814 PMC4059192

[24] Kiger ME, Varpio L. Thematic analysis of qualitative data: AMEE Guide No. 131. Med Teach. 2020;42(8):846–54. 32356468 10.1080/0142159X.2020.1755030

[25] Phillippi J, Lauderdale J. A guide to field notes for qualitative research: Context and conversation. Qual Health Res. 2018;28(3):381–8. 29298584 10.1177/1049732317697102

[26] Blume BD, Kevin Ford J, Surface EA, Olenick J. A dynamic model of training transfer. Hum Resour Manag Rev. 2019;29(2):270–83.

[27] Artioli G, Bedini G, Bertocchi E, Ghirotto L, Cavuto S, Costantini M, Tanzi S. Palliative care training addressed to hospital healthcare professionals by palliative care specialists: A mixed-method evaluation. BMC Palliat Care. 2019;18(1):88. 31655585 10.1186/s12904-019-0476-8 PMC6815393

[28] Dunning D. Chapter five - The Dunning–Kruger Effect: On Being Ignorant of One's Own Ignorance. In: Olson JM, Zanna MP, editors. Advances in Experimental Social Psychology. 44: Academic Press; 2011. p. 247–96.

[29] Back AL. Patient-clinician communication issues in palliative care for patients with advanced cancer. J Clin Oncol. 2020;38(9):866–76. 32023153 10.1200/JCO.19.00128

[30] Liaw SN, Sullivan A, Snaman J, Joselow M, Duncan J, Wolfe J. “We’re performing improvisational jazz”: Interprofessional pediatric palliative care fellowship prepares trainees for team-based collaborative practice. J Pain Symptom Manage. 2021;62(4):768–77. 33600896 10.1016/j.jpainsymman.2021.02.014

[31] Drach LL, Cook M, Shields S, Burger KJ. Changing the culture of pediatric palliative care at the bedside. J Hosp Palliat Nurs. 2021;23(1):20–7. 33136803 10.1097/NJH.0000000000000707

[32] Moynihan KM, Snaman JM, Kaye EC, Morrison WE, DeWitt AG, Sacks LD, et al. Integration of pediatric palliative care into cardiac intensive care: A champion-based model. Pediatrics. 2019;144(2). 10.1542/peds.2019-0160 PMC6855829 31366685

[33] Slater PJ, Herbert AR. Education and mentoring of specialist pediatric palliative care medical and nursing trainees: The Quality of Care Collaborative Australia. Adv Med Educ Pract. 2023;14:43–60. 36726358 10.2147/AMEP.S393051 PMC9885964

[34] Johansen H, Grøndahl VA, Helgesen AK. Palliative care in home health care services and hospitals - the role of the resource nurse, a qualitative study. BMC Palliat Care. 2022;21(1):64. 35501848 10.1186/s12904-022-00956-x PMC9063046

[35] Engel M, van Zuylen L, van der Ark A, van der Heide A. Palliative care nurse champions’ views on their role and impact: A qualitative interview study among hospital and home care nurses. BMC Palliat Care. 2021;20(1):34. 33602177 10.1186/s12904-021-00726-1 PMC7893717

[36] Riggs M, Franklin R, Saylany L. Associations between cardiopulmonary resuscitation (CPR) knowledge, self-efficacy, training history and willingness to perform CPR and CPR psychomotor skills: A systematic review. Resuscitation. 2019;138:259–72. 30928504 10.1016/j.resuscitation.2019.03.019

