# Supplementary material for: Experiences with a national team-based learning program for advance care planning in pediatric palliative care
Source: BMC Palliat Care. 2024 Aug 3;23:196. doi: 10.1186/s12904-024-01515-2 (PMC11297680; doi:10.1186/s12904-024-01515-2)
Supplement: Supplementary file 1 — Supplementary Material 1. [file 12904_2024_1515_MOESM1_ESM.docx]

**Experiences with a national team-based learning program for advance care planning in pediatric palliative care**

**Supplemental file 1**

**The intervention**

A team-based learning program was developed aimed at letting facilitators transfer acquired knowledge and skills and built self-confidence regarding initiating and conducting ACP conversations with their team. The team-based learning program consisted of two elements:

1. **A one-day ‘train-the-trainer’ course** in which facilitators were trained to guide and structure reflection on ACP conversations in their team following a defined structure. Facilitators were trained by researchers that developed IMPACT and have ample experience in training IMPACT (JF and MK). Trained actors (Wilde Kastanje Training and Education, the Netherlands) simulated parents or children in role plays during this course and provided specific feedback on ACP communication skills.

- The first part of the course included the following components and didactic methods:
  - Lecture on the content and structure of IMPACT and explanation of five ACP communication skills: 1. Framing the situation 2. Responding to emotions; 3. Exploring individual perspectives; 4. Giving different perspectives the right to exist; and 5. Achieve a shared point of view and related next steps (1-4)
  - Short role-plays through constructed (given) cases where facilitators practiced the use of IMPACT and ACP communication skills (2)
  - Introduction of a method to facilitate training of ACP communication skills and reflection on ACP conversations during a coaching-on-the-job session in team setting, consisting of setting a learning objective, role-playing and reflecting on experiences. (2, 5)
- The second part focused on training in the role of facilitator and looking forward to the coaching-on-the-job session. It included the following components and didactic methods:
  - Drill the format of the introduced method for practicing and reflection on ACP conversations in teams
  - Short role-plays where facilitators practice this method both as facilitator and learner
  - Instructions for the coaching-on-the-job sessions that facilitators are expected to organize and facilitate in their own PPCT and (practical) agreements
- At the end of the course facilitators prepared a plan of action for the follow-up ‘coaching-on-the-job’ programs in their teams.
- Recap and closure

1. **A coaching-on-the-job program**, ideally consisting of two team sessions of two hours in each PPCT, organized and guided by the newly trained facilitators. A coaching-on-the-job session includes the following components:

- A summary of the knowledge about the use of IMPACT and ACP communication skills in pediatric palliative care
- Short role-plays where team members sequentially practice a self-chosen part of an ACP conversation and a self-chosen ACP communication skill through a real case or a constructed (given) case, as preferred
- The facilitator guides the role-plays using the defined structure that the facilitators had been trained in
- Recap and closure

**References**

1. Back AL, Arnold RM, Tulsky JA, Baile WF, Fryer-Edwards KA. Teaching communication skills to medical oncology fellows. J Clin Oncol. 2003;21(12):2433-6.

2. Back AL, Fromme EK, Meier DE. Training clinicians with communication skills needed to match medical treatments to patient values. J Am Geriatr Soc. 2019;67(S2):S435-s41.

3. Back AL. Patient-clinician communication issues in palliative care for patients with advanced cancer. J Clin Oncol. 2020;38(9):866-76.

4. Fahner J, Rietjens J, van der Heide A, Milota M, van Delden J, Kars M. Evaluation showed that stakeholders valued the support provided by the Implementing Pediatric Advance Care Planning Toolkit. Acta Paediatr. 2021;110(1):237-46.

5. Arnold RM, Back AL, Baile WF, Edwards KA, Tulsky JA. The Oncotalk/Vitaltalk model. In: Kissane DW, Bultz BD, Butow PN, Bylund CL, Noble S, Wilkinson S, editors. Oxford Textbook of Communication in Oncology and Palliative Care: Oxford University Press; 2017. p. 363-8.
