# Supplementary material for: Experiences with a national team-based learning program for advance care planning in pediatric palliative care
Source: BMC Palliat Care. 2024 Aug 3;23:196. doi: 10.1186/s12904-024-01515-2 (PMC11297680; doi:10.1186/s12904-024-01515-2)
Supplement: Supplementary file 2 — Supplementary Material 2. [file 12904_2024_1515_MOESM2_ESM.docx]

**Experiences with a national team-based learning program for advance care planning in pediatric palliative care**

**Supplemental file 2**

**Table S1 Time schedule of enrolment, intervention and data collection**

|  | Study period | | | | | |
| --- | --- | --- | --- | --- | --- | --- |
|  | Month -3  April 2022 | Month -2 | Month 1  August | Month 2 | Month 3 to 5 | Month 6  January 2023 |
| *Enrolment* |  |  |  |  |  |  |
| Field notes | X | X | X |  |  |  |
| Invitations of PPCTs | X |  |  |  |  |  |
| Recruitment of facilitators | X | X |  |  |  |  |
| Kick-off meeting |  | X |  |  |  |  |
| Informed consent facilitators |  |  | X | X |  |  |
|  | | | | | | |
| *Intervention and data collection* |  |  |  |  |  |  |
| Train-the-trainer course |  |  |  | X |  |  |
| Field notes |  |  |  | X | X |  |
| Shortly after the course followed by questionnaire T1 for facilitators |  |  |  | X |  |  |
| Questionnaire T1 for learners |  |  |  | X |  |  |
| Coaching-on-the-job session 1 |  |  |  |  | X |  |
| Field notes |  |  |  |  | X |  |
| Followed by questionnaire T2 for organizing facilitators |  |  |  |  | X |  |
| Followed by questionnaire T2 for participating learners |  |  |  |  | X |  |
| Coaching-on-the-job session 2 |  |  |  |  | X |  |
| Field notes |  |  |  |  | X |  |
| Followed by questionnaire T3 for organizing facilitators |  |  |  |  | X |  |
| Followed by questionnaire T3 for participating learners |  |  |  |  | X |  |
|  | | | | | | |
| End of study period |  |  |  |  |  |  |
| Questionnaire T4 for all facilitators |  |  |  |  |  | X |
| Questionnaire T4 for learners who had at least participated in one coaching-on-the-job session and had filled in at least one previous questionnaire. |  |  |  |  |  | X |
