# Supplementary material for: Experiences with a national team-based learning program for advance care planning in pediatric palliative care
Source: BMC Palliat Care. 2024 Aug 3;23:196. doi: 10.1186/s12904-024-01515-2 (PMC11297680; doi:10.1186/s12904-024-01515-2)
Supplement: Supplementary file 3 — Supplementary Material 3. [file 12904_2024_1515_MOESM3_ESM.docx]

**Supplemental file 3**

**Questionnaires for ‘facilitators’ related to the article**

**“Experiences with a national team-based learning program for advance care planning in pediatric palliative care”**

**Questionnaires for facilitators**

***T1 - Questionnaire for facilitators immediately after the train-the-trainer course***

**Part 1: Demographic and workplace characteristics** **(T1)**

1. What is your age?

- 20-30 years
- 30-40 years
- 40-50 years
- > 50 years

1. Are you

- Male
- Female
- Otherwise/I'd rather not say

1. Do you work as a nurse or as a physician?

- Nurse formally qualified in child care
- Nurse not formally qualified in child care
- Nurse Practitioner
- Medical specialist
- Another function, namely ...

1. For nurses: How many years have you been employed as a registered nurse?
   - 0-5 years
   - 5-10 years
   - Longer than 10 years
2. For physicians: what is your specialty? ......
3. For physicians: how many years have you been working as a physician since obtaining your physician's degree?

- 0-5 years
- 5-10 years
- Longer than 10 years

1. Have you received additional training in the field of palliative care?

- No
- Yes, namely ......

1. Approximately how many children with a life-threatening or life-limiting illness and their parents do you care for each year?

- <5
- 5-10
- 10-20
- >20

1. Have you followed the two-day IMPACT (ACP) training of the Dutch Centre of Expertise in Children's Palliative Care?

- Yes
- No, but I signed up for said ACP training.
- No, I do want to do the ACP training but I haven't registered yet.
- No, I don't know yet if I'm going to do the mentioned training.

**Part 2: Reaction (T1)**

Answer options:

- Not agree at all
- Not agree
- Neutral
- Agree
- Totally agree

1. I found the content of this train-the-trainer course instructive.
2. This train-the-trainer course fitted well with my previously acquired knowledge.
3. I liked the coherence between the parts of the train-the-trainer course.
4. I liked the content of this train-the-trainer course.
5. During the train-the-trainer course I was able to practice enough.
6. The guidance by the trainer was motivating.
7. My trainer gave adequate feedback.
8. The atmosphere during this train-the-trainer course was good.
9. Open question: Which aspects or parts of this train-the-trainer course did you particularly appreciate? Would you like to explain why? ...........
10. Open question: Which aspects or parts of this train-the-trainer course should be changed? Would you like to explain why?

...........

**Part 3: Learning (T1)**

Answer options:

- Not agree at all
- Not agree
- Neutral
- Agree
- Totally agree

1. The train-the-trainer course fits in well with my daily practice.
2. I think the core skills that are needed for conducting an ACP conversation with parents and possibly child are clear.
3. I find the skills needed for methodical reflection on conducting an ACP conversation with parents and possibly child clear.
4. I know what is expected of me in terms of methodical reflection with colleagues on conducting an ACP conversation with parents and possibly child.
5. In my daily practice, I am sufficiently able to transfer the core skills for conducting ACP interviews to colleagues from my PPCT.
6. I am sufficiently able to facilitate methodical reflection on conducting ACP conversations in a team in my PPCT.
7. Open question: What knowledge about conducting ACP conversations has the train-the-trainer course added to your existing knowledge? ..............
8. Open question: How have you become more skilled with regard to conducting ACP conversations with parents and possibly child? ...........
9. Open question: What knowledge about methodically reflecting with colleagues from your Pediatric Palliative Care Team on conducting ACP conversations has the train-the-trainer course added to your already existing knowledge?

………...

**Part 4: Behavior (T1)**

Answer options:

- Not agree at all
- Not agree
- Neutral
- Agree
- Totally agree

1. In my care practice, I regularly reflect on the initiative of someone else with one or more colleagues on preparing for or conducting an ACP conversation with parents and possibly child.
2. In my care practice, I regularly reflect on my initiative with one or more colleagues to prepare for or conduct an ACP conversation with parents and possibly child.
3. In my care practice, I bring up the possibility of an ACP conversation with half or more of the families to whom our Pediatric Palliative Care Team provides care.
4. Open question: Would you like to indicate (an estimate) with how many parents (from 1 family) and possibly child your Pediatric Palliative Care Team as a whole has had an ACP conversation in the past 6 months? …...........
5. Open question: What has changed your attitude and self-confidence as a result of the train-the-trainer course with regard to taking the initiative to methodically reflect on ACP conversations by you or colleagues? Think of the preparation and/or execution of ACP conversations.

..............

1. Which two or three core skills for conducting ACP conversations, which were previously covered in the IMPACT training and recently in the train-the-trainer course, do you think need extra attention in your Pediatric Palliative Care Team?

Answer options:

- Framing the situation
- Explore
- Next steps
- Positioning of conversation partners
- Responding to emotions

1. Open question: Can you explain why you want to work with these three core skills in your Pediatric Palliative Care Team? If possible, explain each core skill you mentioned.

................

1. Open question: What do you fear, what obstacles do you expect when transferring acquired knowledge and skills with regard to conducting ACP interviews to colleagues in your Pediatric Palliative Care Team?

................

1. Open question: What other support do you need to transfer the knowledge and skills you have gained with regard to conducting ACP conversations to your colleagues?

.................

38. Open-ended question: Room for (other) comments or suggestions:

.................

***T2 and T3: Questionnaire for facilitators shortly after the first or second coaching-on-the-job session***

**Part 1: Any changes to workplace characteristics (T2/T3)**

1. Since the train-the trainer course, have you experienced a change in your work situation that affects your role as a facilitator in the VIMP Impact project? Think of: a change in position, a change in activities, a training followed.

- Yes
- No
- Maybe

1. If yes or maybe, would you like to mention this change and state why you think it will impact your role as a facilitator?

....................................

**Part 2: Thoughts now on train-the-trainer course** **(T2/T3)**

1. Open question: How do you look back on the train-the-trainer course? Do you have any additional thoughts about the course? Please explain your answer.

..............

**Part 3: Role of facilitator (T2/T3)**

1. Indicate to what extent you agree with the following statement: 'I was able to successfully implement the plan for organizing the first/second coaching-on-the-job session that I described in response to the train-the-trainer course'.

Answer options:

- Not agree at all
- Not agree
- Neutral
- Agree
- Totally agree

1. [If answer 'Agree' or 'Totally agree']

Open question: Can you describe who or what was helpful in successfully executing your plan of action? Can you elaborate on this?

..............

1. [If answer 'Not agree at all' or 'Not agree' or 'Neutral']

Open question: Who or what would have helped to implement this plan more successfully?

..............

1. In addition to the facilitators, how many professionals participated in the first/second coaching-on-the-job session in your team? Name the number of 'learners'.

..............

1. In your plan of action and in survey 1, you have previously mentioned a number of core skills for conducting ACP conversations that you think need extra attention in your Pediatric Palliative Care Team.

Which core skills for conducting ACP interviews were actually practiced in the coaching-on-the-job session in your Pediatric Palliative Care Team?

Answer options:

- Framing the situation
- Responding to emotions
- Exploring individual perspectives
- Giving different perspectives a right to exist
- Next steps from common ground

1. Open question: To what extent did you take on the role of facilitator in the first coaching-on-the-job session? Can you elaborate on this?

.........................

1. In your action plan, you identified some of the skills for applying methodical reflection in your team that inspired you the most. What skills did you actually apply during the coaching-on-the-job session? In the next question, you will be given space to write about your experiences applying each skill you mentioned.

Answer options (multiple answers possible):

- Determine a learning objective
- Introduce/guide/stop a role-play
- Identify effective strategies
- Discover an area of improvement
- Have replay done/guide/stop
- Identify improvement results
- Summarize the learning experience

1. Open-ended question: Which of the skills for applying methodical reflection in your team that you mentioned in question 10 worked well for you? Can you explain what the situation was, what you said/did and what effect it had on your colleague(s) for one specific skill?

.....................

1. Open-ended question: Which of the skills for applying methodical reflection in your team that you mentioned in question 10 did you find difficult to use? Can you give an example, what happened, would you like to elaborate?

.....................

1. Open-ended question: In your plan of action, you have mentioned expected obstacles and how you could anticipate them. What obstacles did you actually experience when organizing the coaching-on-the-job session, and how did you deal with them?

......................

14. In addition to your role as facilitator, did you also participate in the first coaching-on-the-job session in the role of 'learner'?

- Yes, I participated in the role of facilitator *and* learner.
- No, I only participated in the role of facilitator.
- Other

1. In the previous question about your role during the coaching-on-the-job session, you filled in 'Other'. Can you explain your role during the session? Would you like to indicate which parts of this session you particularly appreciated from your participation role, and which parts need to be changed?

………………………

16. If yes, open question: From your learner perspective, what aspects or parts of this coaching-on-the-job session did you particularly appreciate?

.........................

17. If yes, open question: What aspects or parts of this coaching-on-the-job session should be changed from your learner perspective?

.........................

**Part 4: Required support and final question** **(T2/T3)**

1. Open question: What other support do you need to transfer the knowledge and skills you have gained with regard to conducting ACP conversations to your colleagues?

.................

1. Open question: Room for (other) comments or suggestions:

............

***T4: Questionnaire for facilitators at the end of the study***

**Part 1: Any changes to workplace characteristics (T4)**

1. Since the train-the-trainer course, have you experienced a change in your work situation that affects your role as a facilitator in the VIMP Impact project? Think of: a change in position, a change in activities, a training followed.

- Yes
- No
- Maybe

1. If yes or maybe, would you like to mention this change and state why you think it will impact your role as a facilitator?

....................................

**Part 2: What I have learned (T4)**

Answer options:

- Not agree at all
- Not agree
- Neutral
- Agree
- Totally agree

1. The train-the-trainer course on [date] fitted in well with my daily practice.
2. I think the core skills that are needed for conducting an ACP conversation with parents and possibly child are clear.
3. I find the skills needed for methodical reflection on conducting an ACP conversation with parents and possibly child clear.
4. I know what is expected of me in terms of methodical reflection with colleagues on conducting an ACP conversation with parents and possibly child.
5. In my daily practice, I am sufficiently able to transfer the core skills for conducting ACP interviews to colleagues from my PPCT.
6. I am sufficiently able to facilitate methodical reflection on conducting ACP conversations in a team in my PPCT.

**Part 3: Role of facilitator (T4)**

Please indicate your agreement with the following statements:

Answer options:

- Not agree at all
- Not Agree
- Neutral
- Agree
- Totally agree

1. In my care practice, I regularly reflect on the initiative of someone else with one or more colleagues on preparing for or conducting an ACP conversation with parents and possibly child.
2. In my care practice, I regularly reflect on my initiative with one or more colleagues to prepare for or conduct an ACP conversation with parents and possibly child.
3. In my care practice, I bring up the possibility of an ACP conversation with half or more of the families to whom our Pediatric Palliative Care Team provides care.
4. Even after the end of this VIMP Impact project (which runs until the beginning of February 2023), our team will continue to practice ACP conversations in coaching-on-the-job sessions.
5. If the answer to the previous question was 'Agree' or 'Totally agree': Open-ended question: Can you explain what your plan is to continue practicing ACP conversations in your team and how you plan to do so?

…………………….

1. Open question: To what extent did you take on the role of facilitator in your Pediatric Palliative Care Team in the period after the train-the-trainer course on [date]? Can you elaborate on this?

.........................

1. Open question: Can you indicate whether you do things differently than before when conducting ACP conversations, and if so, what you do differently?

.......................

1. In the train-the-trainer course, you have learned a number of skills for applying methodical reflection in coaching-on-the-job sessions in your team: determine a learning objective, introduce/guide/stop a role-play, identify effective strategies, discover an area of improvement, introduce/guide/stop a replay, identify improvement results, summarize the learning experience. What methodical reflection application skills that you applied during the coaching-on-the-job session(s) do you expect to continue to apply in your team even after the end of this project?

Answer options [multiple answers possible]:

- N/A, there has not been a coaching-on-the-job session in our Pediatric Palliative Care Team.
- N/A, we will not continue to organize coaching-on-the-job or similar sessions to practice ACP conversations.
- Determine a learning objective
- Introduce/guide/stop a role-play
- Identify effective strategies
- Discover an area of improvement
- Introduce/guide/stop the replay
- Identify improvement results
- Summarize the learning experience

1. If the answer to the previous question was 'N/A, there is no coaching in our Pediatric Palliative Care Team' on-the-job session' or 'N/A, we will not continue in our Pediatric Palliative Care Team with organizing coaching-on-the-job or similar sessions to practice ACP conversations': would you like to indicate why you have not practiced these skills or are not continuing to practice ACP conversations in a team setting?

.....................

1. If the answer to the previous question was **not** N/A (answer option 1 or 2): Open-ended question: Can you explain why you continue to use the skills for applying methodical reflection in your team that you mentioned in question 16 in your team?

.....................

1. Open-ended question: Can you explain why you expect to continue to use less of the skills for applying methodical reflection in your team that you ***did not*** mention in question 16? If not applicable, you can fill in: N/A.

.....................

**Part 4: My healthcare practice (T4)**

1. Open question: Would you like to indicate (an estimate) with how many families your Pediatric Palliative Care Team as a whole has had an ACP conversation in the past 6 months?

**.....................**

1. Indicate to what extent you agree with the following statement: 'I have achieved the goal I set in my plan of action that I wanted to achieve with the knowledge and skills provided for methodical reflection in the team' .

Answer options:

- Not agree at all
- Not agree
- Neutral
- Agree
- Totally agree

1. [If answer 'Not agree at all' or 'Not agree' or 'Neutral']

Open question: Can you explain what you think are the main reasons why you have not (partially) achieved the goal you have set?

......................

1. [If answer 'Agree' or 'Totally agree']

Open question: Can you describe what shows that you have achieved the goal of your action plan and what has contributed to it? Can you elaborate on this?

..............

1. Open question: What has changed your attitude and self-confidence as a result of the train-the-trainer course with regard to taking the initiative to methodically reflect on ACP conversations by you or colleagues? Think of reflecting and/or practising the preparation and/or conducting of ACP conversations.

......................

1. Please indicate to what extent you agree with the following statement: I expect that I will continue to apply the skills learned for methodical reflection in my Pediatric Palliative Care Team after the end of this research period.

Answer options:

- Not agree at all
- Not Agree
- Neutral
- Agree
- Totally agree

1. Have you planned your next coaching-on-the-job session in your Pediatric Palliative Care Team?

Answer options:

- Yes, the date is already known.
- Yes, but no date has been announced yet.
- No, but I'm going to schedule an on-the-job coaching session.
- No, I don't know if I'm going to schedule an on-the-job coaching session (again).
- No, I'm not going to schedule another coaching-on-the-job session.

**Part 5: General questions and final question (T4)**

1. Open-ended question: In your plan of action, you have mentioned expected obstacles and how you could anticipate them. What obstacles have you actually experienced throughout the entire process since the train-the-trainer course, and how have you dealt with them?

........................

1. Which aspects or parts of the entire train-the-trainer course and coaching-on-the-job process (from the course on [date] until now) have you particularly appreciated?

.........................

1. Open question: Which aspects or parts of the entire train-the-trainer and coaching-on-the-job trajectory (from the course on [date]) should be changed so far? Would you like to explain why?

.........................

1. Open question: Do you have any additional comments or suggestions?

.........................

*We would like to thank you very much for filling out the questionnaire!*
