# Supplementary material for: Experiences with a national team-based learning program for advance care planning in pediatric palliative care
Source: BMC Palliat Care. 2024 Aug 3;23:196. doi: 10.1186/s12904-024-01515-2 (PMC11297680; doi:10.1186/s12904-024-01515-2)
Supplement: Supplementary file 4 — Supplementary Material 4. [file 12904_2024_1515_MOESM4_ESM.docx]

**Supplemental file 4**

**Questionnaires for ‘learners’ related to the article**

**“Experiences with a national team-based learning program for advance care planning in pediatric palliative care”**

***T1 - Questionnaire for learners at the start of the study***

**Part 1: Demographic and workplace characteristics (T1)**

1. What is your age?

- 20-30 years
- 30-40 years
- 40-50 years
- > 50 years

1. Are you

- Male
- Female
- Otherwise/I'd rather not say

1. Do you work as a nurse or as a doctor?

- Nurse formally qualified in child care
- Nurse not formally qualified in child care
- Medical specialist
- Another function, namely ...

1. For nurses: How many years have you been working as a registered nurse?
   - 0-5 years
   - 5-10 years
   - Longer than 10 years
2. For physicians: what is your specialty? ......
3. For physicians: how many years have you been working as a physicians since obtaining your physicians's degree?

- 0-5 years
- 5-10 years
- Longer than 10 years

1. Have you received additional training in the field of palliative care?

- No
- Yes, namely ......

1. Approximately how many children with a life-threatening or life-limiting illness and their parents do you care for each year?

- <5
- 5-10
- 10-20
- >20

1. Have you followed the two-day IMPACT (ACP) training of the Dutch Centre of Expertise in Children's Palliative Care?

- Yes
- No, but I signed up for said ACP training.
- No, I do want to do the ACP training but I haven't registered yet.
- No, I don't know yet if I'm going to do the mentioned training.

**Part 2: Attitudes and beliefs towards Advance Care Planning (ACP) (T1)**

Answer options:

- Not agree at all
- Not agree
- Neutral
- Agree
- Totally agree

1. I feel comfortable preparing parents for an ACP conversation.
2. I feel comfortable preparing a child for an ACP conversation.
3. I feel comfortable having ACP conversations with parents.
4. I feel comfortable having an ACP conversation with a child.

15. I have sufficient knowledge about how to conduct ACP conversations with parents and possibly the child.

1. Conducting ACP conversations with parents of a child with a life-threatening or life-limiting illness and, depending on the age, the child, is my professional responsibility.
2. Conducting ACP conversations with parents and possibly the child can improve the care for these parents and the child.

**Part 3: Behavior (T1)**

Answer options:

- Not agree at all
- Not agree
- Neutral
- Agree
- Totally agree

1. In my care practice, I regularly reflect on the initiative of someone else with one or more colleagues on preparing for or conducting an ACP conversation with parents and possibly child.
2. In my care practice, I regularly reflect on my initiative with one or more colleagues to prepare for or conduct an ACP conversation with parents and possibly child.
3. In my care practice, I bring up the possibility of an ACP conversation with half or more of the families to whom our Pediatric Palliative Care Team provides care.
4. Open question: Would you like to indicate (an estimate) with how many parents (from 1 family) and possibly child your Pediatric Palliative Care Team as a whole has had an ACP conversation in the past 6 months? ............
5. Would you like to indicate (an estimate) with how many parents (from 1 family) and possibly child you have had an ACP conversation in the past 6 months? ............
6. Open question: What do you think you do very well in conducting ACP conversations with parents? ...............
7. Open question: What do you think you do very well in conducting ACP conversations with a child? ...............
8. Open question: What do you find difficult when conducting ACP conversations with parents?

...............

1. Open question: What do you find difficult when conducting ACP conversations with a child?

...............

1. Open question: What support would you like to gain more knowledge and skills with regard to conducting ACP conversations?

.................

1. Open question: Room for (other) comments or suggestions:

............

***T2 and T3: Questionnaire for learners shortly after the first or second coaching-on-the-job session***

**Part 1: Any changes to workplace characteristics (T2/T3)**

**[For learners who completed survey T1 for learners.**

**For those who *did not* complete questionnaire 1, we made a separate version of questionnaire T2 and T3 containing all previous questions on 'Demographic and workplace characteristics' from T1.]**

1. Has there been a change in your work situation since mid-September 2022 that affects how you conduct ACP conversations in your work? Think of: a change in position, a change in activities, a training followed.

- Yes
- No
- Maybe

1. If yes or maybe, would you like to mention this change and state why you think it will affect the conduct of ACP conversations?

....................................

**Part 2: Attitudes and beliefs towards Advance Care Planning (ACP) (T2/T3)**

**This part we only asked those learners who *did not* complete questionnaire 1. We made a separate version of questionnaire T2 and T3 containing all previous questions on ‘Attitudes and Beliefs towards Advance Care Planning’ from T1.]**

**Part 3: Evaluation of the coaching-on-the-job session (T2/T3)**

Answer options:

- Not agree at all
- Not agree
- Neutral
- Agree
- Totally agree

1. The purpose of this coaching-on-the-job session was clear to me.
2. The set-up of this coaching-on-the-job session was clear.
3. I found the content of this coaching-on-the-job session instructive.
4. This coaching-on-the-job session fitted in well with my previously acquired knowledge.
5. I liked the coherence between the components of the coaching-on-the-job session.
6. I liked the content of this coaching-on-the-job session.
7. During the coaching-on-the-job session I had/practiced the following role(s):

Answer options (multiple answers possible):

- Professional
- Parent
- Observator
- Co-writer

1. During the coaching-on-the-job session, I was able to practice enough.
2. The guidance by the facilitator was motivating.
3. My facilitator provided adequate feedback.
4. The atmosphere during this coaching-on-the-job session was good.
5. The atmosphere during this coaching-on-the-job session was safe.
6. Open question: What aspects or parts of this coaching-on-the-job session did you particularly appreciate? Would you like to explain why?

...........

1. Open question: Which aspects or parts of this coaching-on-the-job session should be changed? Would you like to explain why?

...........

**Part 4: What I learned from the coaching-on-the-job session (T2/T3)**

Answer options:

- Not agree at all
- Not agree
- Neutral
- Agree
- Totally agree

1. The coaching-on-the-job session fits in well with my daily practice.
2. I think the core skills that are needed for conducting an ACP conversation with parents and possibly child are clear.
3. I find the added value of methodically reflecting on conducting an ACP conversation with parents and possibly child with the help of a role play clear.
4. Open question: What knowledge about conducting ACP conversations has the coaching-on-the-job session added to your already existing knowledge? ..............
5. Open question: How did participating in the coaching-on-the-job session make you more proficient in conducting ACP conversations with parents and possibly child? … ...........
6. To what extent did you notice that those who led the session in your team (facilitators) followed a fixed structure?

- Yes
- No
- Unclear

1. (Open-ended question) If so, what did you think of that structure?

................

**Part 5: Healthcare practice (T2/T3)**

Answer options:

- Not agree at all
- Not agree
- Neutral
- Agree
- Totally agree

1. Open question: To what extent has the coaching-on-the-job session changed anything in your attitude and self-confidence with regard to conducting ACP conversations? Can you name one specific learning moment that you experienced as very powerful during the coaching-on-the-job session? Can you explain what happened and why you found this to be a powerful learning opportunity?

...............

1. Open question: What support would you like to gain more knowledge and skills with regard to conducting ACP conversations?

.................

1. Open question: Room for (other) comments or suggestions:

............

***T4: Questionnaire for learners at the end of the study***

**Part 1: Any changes to workplace characteristics (T4)**

1. Has there been a change in your work situation since you completed the previous questionnaire about conducting ACP conversations that affects how you conduct ACP conversations in your work? Think of: a change in position, a change in activities, a training followed.

- Yes
- No
- Maybe

1. If yes or maybe, would you like to mention this change and state why you think it will affect the conduct of ACP conversations?

....................................

**Part 2: What I have learned (T4)**

Please indicate your agreement with the following statements:

Answer options:

- Not agree at all
- Not agree
- Neutral
- Agreee
- Totally agree

1. I feel comfortable preparing parents for an ACP conversation.
2. I feel comfortable preparing a child for an ACP conversation.
3. I feel comfortable having ACP conversations with parents.
4. I feel comfortable having an ACP conversation with a child.
5. I have sufficient knowledge about how to conduct an ACP conversation with parents and/or child.
6. The coaching-on-the-job session(s) I attended fitted in well with my daily practice.
7. I find the core skills that are needed for conducting an ACP conversation with parents and/or child clear.
8. I find the added value of methodically reflecting on having an ACP conversation with parents and/or child with the help of a role play clear.
9. Open question: What knowledge about conducting ACP conversations has the coaching-on-the-job session(s) added to your existing knowledge? ..............
10. Please indicate to what extent you agree with the following statement: During the coaching-on-the-job sessions, the learning points from the 'replay' of the role play were clear to me.

Answer options:

- Not agree at all
- Not agree
- Neutral
- Agree
- Totally agree

1. Open question: How did participating in the coaching-on-the-job session(s) make you more proficient in conducting ACP conversations with parents and possibly child? ...........
2. To what extent did you notice that those who led the coaching-on-the-job session(s), in which ACP conversations were practiced, followed a fixed structure in your team (facilitators)?

- Yes
- No
- Unclear

1. (Open-ended question) If so, do you think that structure is suitable to use more often for practicing ACP conversations? Could you explain your answer?

................

**Part 3: What I bring to my daily practice (T4)**

Please indicate your agreement with the following statements:

Answer options:

- Not agree at all
- Not agree
- Neutral
- Agree
- Totally agree

1. In my care practice, I regularly reflect on the initiative of someone else with one or more colleagues on preparing for or conducting an ACP conversation with parents and possibly child.
2. In my care practice, I regularly reflect on my initiative with one or more colleagues to prepare for or conduct an ACP conversation with parents and possibly child.
3. In my care practice, I bring up the possibility of an ACP conversation with half or more of the families to whom our Pediatric Palliative Care Team provides care.
4. Can you indicate which core ACP conversation skills, which were covered in the coaching-on-the-job session(s), you feel more proficient after practicing in that session? [more answers possible]

Answer options:

- Framing the situation
- Responding to emotions
- Exploring individual perspectives
- Giving different perspectives a right to exist
- Next steps from common ground

1. Can you indicate which core skills for conducting ACP conversations you have actually started to use more  *in ACP conversations with parents and/or child after practicing in a coaching-on-the-job session*? [more answers possible]

Answer options:

- Framing the situation
- Responding to emotions
- Exploring individual perspectives
- Giving different perspectives a right to exist
- Next steps from common ground

1. Can you indicate in which core skills for conducting ACP conversations do you feel as skilled or incompetent as before after practicing in an on-the-job coaching-session? [more answers possible]

Answer options:

- Framing the situation
- Responding to emotions
- Exploring individual perspectives
- Giving different perspectives a right to exist
- Next steps from common ground

1. Open question: If you do things differently in ACP conversations than before, can you explain what you have started to do differently in terms of conducting ACP conversations after participating in the coaching-on-the-job session(s)?

.......................

1. Open question: Can you indicate whether you missed anything in the coaching-on-the-job session(s) about practicing ACP conversations that would be helpful for you in your daily work?

........................

**Part 4: My healthcare practice (T4)**

1. Open question: Would you like to indicate (an estimate) with how many families your Pediatric Palliative Care Team as a whole has had an ACP conversation in the past 6 months?

**.....................**

1. Has the coaching-on-the-job session(s) changed anything in your attitude and self-confidence with regard to ACP conversations with parents and/or child by you or colleagues? Think of the preparation and/or execution of ACP conversations.

- Yes
- No
- Maybe

1. If so, or perhaps, would you please explain what has changed and what that means?

....................................

1. Please indicate to what extent you agree with the following statement: I expect that I will continue to apply the core skills I have learned for conducting ACP conversations during ACP conversations after the end of this research period.

Answer options:

- Not agree at all
- Not agree
- Neutral
- Agree
- Totally agree

1. Please indicate to what extent you agree with the following statement: I am determined to participate in the event that a next session is planned in my Pediatric Palliative Care Team for practicing conducting ACP conversations.

Answer options:

- Not agree at all
- Not agree
- Neutral
- Agree
- Totally agree

**Part 5: General questions and final question (T4)**

1. Which aspects or parts of the entire on-the-job coaching process did you particularly appreciate?

.........................

1. Open question: Which aspects or parts of the entire coaching-on-the-job process should be changed? Would you like to explain why?

.........................

1. Open question: Do you have any additional comments or suggestions?

.........................

*We would like to thank you very much for filling out the questionnaire!*
