# Supplementary material for: Experiences with a national team-based learning program for advance care planning in pediatric palliative care
Source: BMC Palliat Care. 2024 Aug 3;23:196. doi: 10.1186/s12904-024-01515-2 (PMC11297680; doi:10.1186/s12904-024-01515-2)
Supplement: Supplementary file 5 — Supplementary Material 5. [file 12904_2024_1515_MOESM5_ESM.docx]

**Experiences with a national team-based learning program for advance care planning in pediatric palliative care**

**Supplemental file 5**

**Table S1 Satisfaction with training activities in the team-based learning program**

| **Satisfaction with the train-the-trainer course, by facilitators (T1)** | | | |
| --- | --- | --- | --- |
| **Statements** | **(Totally) agree** | **Neutral** | **Not agree (at all)** |
|  | Total  (N = 18)  N (%) | Total  (N = 18)  N (%) | Total  (N = 18)  N (%) |
| I found the content of this train-the-trainer course instructive.^a^ | 18 (100.0) | 0 (0.0) | 0 (0.0) |
| This train-the-trainer course was well suited to my daily practice.^a^ | 15 (83.3) | 3 (16.7) | 0 (0.0) |
| The coherence between the components of the train-the-trainer course was good.^a^ | 18 (100.0) | 0 (0.0) | 0 (0.0) |
| I liked the build-up of content in this train-the-trainer course.^a^ | 17 (94.4) | 1 (5.6) | 0 (0.0) |
| During the train-the-trainer course I was able to practice sufficiently.^a^ | 17 (94.4) | 1 (5.6) | 0 (0.0) |
| The guidance by the trainer was motivating.^a^ | 18 (100.0) | 0 (0.0) | 0 (0.0) |
| My trainer gave adequate feedback.^a^ | 18 (100.0) | 0 (0.0) | 0 (0.0) |
| The general atmosphere during this train-the-trainer course was good.^a^ | 18 (100.0) | 0 (0.0) | 0 (0.0) |
|  | | | |
| **Satisfaction with the coaching-on-the-job session(s), by learners^b^ (T2/T3)** | | | |
| **Statements** | **(Totally) agree** | **Neutral** | **Not agree (at all)** |
|  | Total  (N = 34)  N (%) | Total  (N = 34)  N (%) | Total  (N = 34)  N (%) |
| The purpose of this of this coaching-on-the-job session was clear to me.^a^ | 31 (91.2) | 1 (2.9) | 2 (5.9) |
| The structure of this coaching-on-the-job session was clear.^a^ | 31 (91.2) | 1 (2.9) | 2 (5.9) |
| I found the content of this coaching-on-the-job session instructive.^a^ | 32 (94.1) | 2 (5.9) | 0 (0.0) |
| This coaching-on-the-job session well matched with my previously acquired knowledge.^a^ | 31 (91.2) | 3 (8.8) | 0 (0.0) |
| The coherence between the components of the coaching-on-the-job session was good.^a^ | 27 (79.4) | 7 (20.6) | 0 (0.0) |
| I liked the build-up of content in this coaching-on-the-job session.^a^ | 30 (88,2) | 4 (11.8) | 0 (0.0) |
| During the coaching-on-the-job session I was able to practice sufficiently.^a^ | 24 (70.6) | 6 (17.6) | 4 (11.8) |
| The guidance by the facilitator was motivating.^a^ | 34 (100.0) | 0 (0.0) | 0 (0.0) |
| My facilitator gave adequate feedback.^a^ | 32 (94.1) | 2 (5.9) | 0 (0.0) |
| The general atmosphere during this coaching-on-the-job session was good.^a^ | 34 (100.0) | 0 (0.0) | 0 (0.0) |
| The general atmosphere during this coaching-on-the-job session was safe.^a^ | 34 (100.0) | 0 (0.0) | 0 (0.0) |

a: Participants could answer on a five-point scale ‘totally agree’/‘agree’/‘neutral’/‘not agree’/‘not agree at all’. Totally agree/agree answers and not agree/not agree at all answers were combined in this table.

b: In total, 28 learners participated in one (n = 22) or two (n = 6) coaching-on-the-job sessions, and reported in total on 34 learner experiences.

**Table S2 What professionals learned from the training program**

| **Knowledge and skills learned in the train-the-trainer course, as reported by facilitators (T1 and T4)** | | | | | | |
| --- | --- | --- | --- | --- | --- | --- |
| **Statements** | **(Totally) agree** | | **Neutral** | | **Not agree (at all)** | |
|  | **Question-naire T1^a^**  **N=18 (100.0)** | **Question-naire T4^b^**  **N=14 (100.0)** | **Question-naire T1**  **N=18 (100.0)** | **Question-naire T4**  **N=14**  **(100.0)** | **Question-naire T1**  **N=18**  **(100.0)** | **Question-naire T4**  **N=14**  **(100.0)** |
| The train-the-trainer course aligns well with my previous knowledge.^c^ | 18 (100.0) | 13 (92.9) | 0 (0.0) | 1 (7.1) | 0 (0.0) | 0 (0.0) |
| I find the core skills needed for conducting an ACP conversation with parents and/or child clear.^c^ | 17 (94.4) | 14 (100.0) | 0 (0.0) | 0 (0.0) | 1 (5.6) | 0 (0.0) |
| I find the skills needed for methodically reflecting on conducting an ACP conversation with parents and/or child in team context clear.^c^ | 18 (100.0) | 14 (100.0) | 0 (0.0) | 0 (0.0) | 0 (0.0) | 0 (0.0) |
| I know what is expected of me regarding methodically reflecting with colleagues on conducting an ACP conversation with parents and/or child.^c^ | 15 (83.3) | 12 (85.7) | 3 (16.7) | 2 (14.3) | 0 (0.0) | 0 (0.0) |
| I am sufficiently able to transfer in my daily practice the core skills for conducting ACP conversations to colleagues in my PPCT.^c^ | 8 (44.4) | 9 (64.3) | 9 (50.0) | 4 (28.6) | 1 (5.6) | 1 (7.1) |
| I am sufficiently able to facilitate methodical reflection on conducting ACP conversations in team settings in my PPCT.^c^ | 9 (50.0) | 8 (57.1) | 8 (44.4) | 4 (28.6) | 1 (5.6) | 2 (14.3) |
|  | **Questionnaire T4** | | | | | **N =14**  **N (%)** |
| Skills for practicing and methodical reflection on ACP conversations that facilitators applied during the coaching-on-the-job session(s) and that they expect to continue to use, even after the completion of this study (mentioned by n (%)) | Determine learning objective | | | | | 10 (71.4) |
|  | Introduce/guide/stop role play | | | | | 11 (78.6) |
|  | Identify effective strategies | | | | | 10 (71.4) |
|  | Discover area of improvement | | | | | 10 (71.4) |
|  | Introduce/guide/stop the replay | | | | | 10 (71.4) |
|  | Identify improvement results | | | | | 8 (57.1) |
|  | Summarize learning experience | | | | | 9 (64.3) |
|  | Not applicable, there was no coaching-on-the-job session in our PPCT | | | | | 2 (14.3) |

|  | | | | | | |
| --- | --- | --- | --- | --- | --- | --- |
| **Knowledge and skills learned in the coaching-on-the-job session(s), as reported by learners (T1 to T4)** | | | | | | |
| **Statements** | **(Totally) agree** | | **Neutral** | | **Not agree (at all)** | |
|  | **Question-naire T1^d^**  **N=31**  **(100.0)** | **Question-naire T4^e^**  **N=21**  **(100.0)** | **Question-naire T1**  **N=31**  **(100.0)** | **Question-naire T4**  **N=21**  **(100.0)** | **Question-naire T1**  **N=31**  **(100.0)** | **Question-naire T4**  **N=21**  **(100.0)** |
| I feel comfortable preparing parents for an ACP conversation.^c^ | 23 (74.2) | 14 (66,7) | 6 (19.4) | 7 (33.3) | 2 (6.5) | 0 (0.0) |
| I feel comfortable preparing a child for an ACP conversation.^c^ | 12 (38.7) | 10 (47.6) | 13 (41.9) | 10 (47.6) | 6 (19.4) | 1 (4.8) |
| I feel comfortable conducting ACP conversations with parents.^c^ | 20 (64.5) | 13 (61.9) | 8 (25.8) | 8 (38.1) | 3 (9.7) | 0 (0.0) |
| I feel comfortable conducting ACP conversations with a child.^c^ | 13 (41.9) | 11 (52.4) | 10 (32.3) | 9 (42.9) | 8 (25.8) | 1 (4.8) |
| I have sufficient knowledge about how to conduct ACP conversations with parents and/or the child.^c^ | 13 (41.9) | 13 (61.9) | 13 (41.9) | 6 (28.6) | 5 (16.1) | 2 (9.5) |
|  | **Question-naire T2 and T3^f^**  **N=34**  **(100.0)** | **Question-naire T4^e^**  **N=21**  **(100.0)** | **Question-naire T2 and T3**  **N=34**  **(100.0)** | **Question-naire T4**  **N=21**  **(100.0)** | **Question-naire T2 and T3**  **N=34**  **(100.0)** | **Question-naire T4**  **N=21**  **(100.0)** |
| The coaching-on-the-job session(s) I attended aligns well with my daily practice.^c^ | 30 (88.2) | 16 (67.2) | 4 (11.8) | 5 (23.8) | 0 (0.0) | 0 (0.0) |
| I find the core skills needed to conduct an ACP conversation with parents and/or child clear.^c^ | 30 (88.2) | 20 (95.2) | 4 (11.8) | 1 (4.8) | 0 (0.0) | 0 (0.0) |
| I find the added value of methodically reflecting on conducting an ACP conversation with parents and/or child with the help of a role play clear.^c^ | 32 (94.1) | 19 (90.5) | 2 (5.9) | 2 (9.5) | 0 (0.0) | 0 (0.0) |

a: Questionnaire T1 refers to the answers that facilitators gave in the first questionnaire/questionnaire T1 (FAC version) shortly after the train-the-trainer course.

b: Questionnaire T4 refers to the answers that a total of 14 facilitators gave in the last questionnaire/questionnaire T4 (FAC version).

c: Participants could answer on a five-point scale ‘totally agree’/‘agree’/‘neutral’/‘not agree’/‘not agree at all’. Totally agree/agree answers and not agree/not agree at all answers were combined in this table.

d: Questionnaire T1 refers to the answers that a total of 31 learners gave in the first questionnaire/ questionnaire T1 (LEARNER version).

e: Questionnaire T4 refers to the answers that a total of 21 learners gave in the last questionnaire /questionnaire T4 (LEARNER version).

f: Questionnaire T2 and T3 refer to the answers that reflect a total of 34 learner experiences reported in questionnaire T2 and T3, combined, provided by 28 individual learners attending one (n = 22) or two (n = 6) coaching-on-the-job sessions (LEARNER version).

**Table S3 Professionals’ behavior in the clinical setting**

| **Behavior in the clinical setting, as reported by facilitators (T1 to T4)** | | | | | | |
| --- | --- | --- | --- | --- | --- | --- |
|  | **Questionnaire T2 after coaching-on-the-job session 1**  **N = 16 (100.0)^a^** | | | | | |
|  | **(Totally) agree** | | **Neutral** | | **Not agree (at all)** | |
| I was able to successfully execute my plan for organizing the first coaching-on-the-job session which I wrote during the train-the-trainer course.^b^ | 11 (68.8) | | 3 (18.8) | | 2 (12.5) | |
|  | **Questionnaire T3 after coaching-on-the-job session 2**  **N = 8 (100.0)^c^** | | | | | |
| I was able to successfully execute my plan for organizing the second coaching-on-the-job session which I wrote during the train-the-trainer course.^b^ | 7 (87.5) | | 1 (12.5) | | 0 (0.0) | |
|  | **Question-naire T1^d^**  **N=18 (100.0)** | **Question-naire T4^e^**  **N=14**  **(100.0)** | **Question-naire T1**  **N=18**  **(100.0)** | **Question-naire T4**  **N=14**  **(100.0)** | **Question-naire T1**  **N=18**  **(100.0)** | **Question-naire T4**  **N=14**  **(100.0)** |
| In my clinical setting, I regularly reflect on another person's initiative with one or more colleagues on preparing for or conducting an ACP conversation with parents and/or child.^b^ | 6 (27.8) | 5 (35.7) | 8 (44.4) | 4 (28.6) | 4 (22.2) | 5 (35.7) |
| In my clinical setting, I regularly reflect on my initiative with one or more colleagues on preparing for or conducting an ACP conversation with parents and/or child.^b^ | 8 (44.4) | 10 (71.4) | 6 (33.3) | 3 (21.4) | 4 (22.2) | 1 (7.1) |
| In my clinical setting, I raise the opportunity of an ACP conversation with half or more of the families to whom our PPCT provides care.^b^ | 11 (61.1) | 10 (71.4) | 2 (11.1) | 2 (14.3) | 5 (27.8) | 2 (14.3) |
|  | | | | | | |

| **Behavior in the clinical setting, as reported by learners (T1 and T4)** | | | | | | | |
| --- | --- | --- | --- | --- | --- | --- | --- |
|  | **(Totally) agree** | | **Neutral** | | | **Not agree (at all)** | |
|  | **Question-naire T1^f^**  **N=31**  **(100.0)** | **Question-naire T4^g^**  **N=21**  **(100.0)** | **Question-naire T1**  **N=31**  **(100.0)** | **Question-naire T4**  **N=21**  **(100.0)** | | **Question-naire T1**  **N=31**  **(100.0)** | **Question-naire T1**  **N=21**  **(100.0)** |
| In my clinical setting, I regularly reflect on another person's initiative with one or more colleagues on preparing for or conducting an ACP conversation with parents and/or child.^b^ | 16 (51.6) | 13 (61.9) | 9 (29.0) | 7 (33.3) | | 6 (19.4) | 1 (4.8) |
| In my clinical setting, I regularly reflect on my initiative with one or more colleagues on preparing for or conducting an ACP conversation with parents and/or child.^b^ | 15 (48.4) | 13 (61.9) | 10 (32.3) | 6 (28.6) | | 6 (19.4) | 2 (9.5) |
| In my clinical setting, I raise the possibility of an ACP conversation with half or more of the families to whom our PPCT provides care.^b^ | 13 (41.9) | 13 (61.9) | 8 (25.8) | 6 (28.6) | | 10 (32.3) | 2 (9.5) |
|  | **Questionnaire T4^g^**  **N=21 (100.0)** | | | | | | |
| Core skills for conducting ACP conversations, previously addressed in the coaching-on-the-job session(s) attended, in which one feels more competent after practicing in that session, mentioned by number of learners (More answers possible) | Framing the situation  Responding to emotions  Exploring perspectives  Giving different perspectives the right to exist  Next steps from common ground | | | | 9 (42.9)  10 (47.6)  10 (47.6)  12 (57.1)  7 (33.3) | | |
| Core skills for conducting ACP conversations, previously addressed in the coaching-on-the-job session, that one has actually started to use more in ACP conversations with parents and/or child after practicing with them, mentioned by number of learners (More answers possible) | Framing the situation  Responding to emotions  Exploring perspectives  Giving different perspectives the right to exist  Next steps from common ground | | | | 5 (23.8)  6 (28.6)  6 (28.6)  7 (33.3)  7 (33.3) | | |

a: In total, 16 facilitators from 7 PPCTs organized and facilitated at least a first coaching-on-the-job session.

b: Participants could answer on a five-point scale ‘totally agree’/‘agree’/‘neutral’/‘not agree’/‘not agree at all’. Totally agree/agree answers and not agree/not agree at all answers were combined in this table.

c: In total, 8 facilitators from 4 PPCTs organized and facilitated a second coaching-on-the-job session.

d: Questionnaire T1 refers to the answers that facilitators gave in questionnaire T1 (FAC version) shortly after the train-the-trainer course.

e: Questionnaire T4 refers to the answers that a total of 14 facilitators gave in questionnaire T4 (FAC version).

f: Questionnaire T1 refers to the answers that a total of 31 learners gave in questionnaire T1 (LEARNER version).

g: Questionnaire T4 refers to the answers that a total of 21 learners gave in questionnaire T4 (LEARNER version).

**Table S4 Results regarding practicing with and reflecting on ACP conversations in PPCTs**

| **Results in their PPCT, as reported by facilitators (T1 and T4)** | | | | | | |
| --- | --- | --- | --- | --- | --- | --- |
|  | **Question- naire T1^a^**  **N=18**  **(100.0)** | **Question-naire T4^b^**  **N=14**  **(100.0)** | **Question-naire T1**  **N=18**  **(100.0)** | **Question-naire T4**  **N=14**  **(100.0)** | **Question-naire T1**  **N=18**  **(100.0)** | **Question-naire T4**  **N=14**  **(100.0)** |
|  | 0-10 families | | 11-20 families | | No number mentioned/ 'I don't know'. | |
| Number of families (an estimate) with whom my PPCT as a whole has had an ACP conversation in the past 6 months (open question) | 9 (50.0) | 5 (35.7) | 4 (22.2) | 5 (35.7) | 5 (27.8) | 4 (28.6) |
|  | | | | | | |
| The following statements/questions were presented in the final questionnaire T4. | **Questionnaire T4^b^**  **N = 14 (100.0)** | | | | | |
|  | **(Totally) agree** | | **Neutral** | | **Not agree (at all)** | |
| I have met the goal that I wanted to achieve with the knowledge and skills taught for methodical reflection in the team that I set at the end of the train-the-trainer course in my action plan.^c^ | 7 (50.0) | | 4 (28.6) | | 3 (21.4) | |
| I expect to continue to apply the skills learned for methodical reflection in my PPCT beyond the end of this research period.^c^ | 12 (85.7) | | 1 (7.1) | | 1 (7.1) | |
| Even after the end of this VIMP Impact project (which runs until early February 2023), our PPCT will continue to practice ACP conversations in coaching-on-the-job sessions.^c^ | 11 (78.6) | | 3 (21.4) | | 0 (0.0) | |
|  | **Open answers grouped by category** | | | | | **N** |
| How has the train-the-trainer course changed your attitude and self-confidence toward taking the initiative to methodically reflect on ACP conversations by you or colleagues? (Open question) | Self-confidence in the facilitator role has grown/is growing.  Self-confidence in conducting ACP conversations has grown/growing.  I have more experience, knowledge about theory.  Nothing changed in basic attitude, but I see more connection in the team.  Nothing changed in my attitude and/or self-confidence. | | | | | 6  3  1  1  3 |
|  | **Yes, the date is already known** | | **No, but I will** | | **I don't know yet/I'm not going to do that** | |
| I have scheduled another coaching-on-the-job session in my PPCT. | 2 (14.3) | | 9 (64.3) | | 3 (21.4) | |
|  | | | | | | |
| **Results in their PPCT, as reported by learners (T1 and T4)** | | | | | | |
|  | **Question-naire T1^d^**  **N=31**  **(100.0)** | **Question-naire T4^e^**  **N=21**  **(100.0)** | **Question-naire T1**  **N=31**  **(100.0)** | **Question-naire T4**  **N=21**  **(100.0)** | **Question-naire T1**  **N=31**  **(100.0)** | **Question-naire T4**  **N=21**  **(100.0)** |
|  | 0-10 families | | 11-20 families | | No number mentioned/ 'I don't know'. | |
| Number of families (an estimate) with whom my PPCT as a whole has had an ACP conversation in the past 6 months (open question) | 14 (45.2) | 13 (61.9) | 10 (32.3) | 4 (19.0) | 7 (22.6) | 4 (19.0) |
|  | | | | | | |
| The following statements/questions were presented in the final questionnaire T4. | **Questionnaire T4^e^**  **N=21 (100.0)** | | | | | |
|  | **Yes** | | **No** | | **Maybe** | |
| Did the coaching-on-the-job session(s) that you attended change anything in your attitude and self-confidence toward conducting ACP interviews by you or colleagues? | 10 (47.6) | | 5 (23.8) | | 6 (28.6) | |
|  | **(Totally) agree** | | **Neutral** | | **Not agree (at all)** | |
| I expect to continue to apply the core ACP interviewing skills learned during ACP conversations after the end of this research period.^c^ | 18 (85.7) | | 3 (14.3) | | 0 (0.0) | |
| I strongly intend that if a subsequent session for practicing ACP conversations is scheduled in my PPCT, I will participate in it.^c^ | 15 (71.4) | | 5 (23.8) | | 1 (4.8) | |

a: Questionnaire T1 refers to the answers that facilitators gave in questionnaire T1 (FAC version) shortly after the train-the-trainer course.

b: Questionnaire T4 refers to the answers that a total of 14 facilitators gave in questionnaire T4 (FAC version).

c: Participants could answer on a five-point scale ‘totally agree’/‘agree’/‘neutral’/‘not agree’/‘not agree at all’. Totally agree/agree answers and not agree/not agree at all answers were combined in this table.

d: Questionnaire T1 refers to the answers that a total of 31 learners gave in questionnaire T1 (LEARNER version).

e: Questionnaire T4 refers to the answers that a total of 21 learners gave in questionnaire T4 (LEARNER version).
